# A Giving Pledge of sharing discovery

**DOI:** 10.1039/c6sc90079h

**Published:** 2016-12-08

## Abstract

Editor-in-Chief Dan Nocera encourages the chemical science community to share the wealth of their most exceptional scientific discoveries, so that this is freely available to all.

## Abstract 2

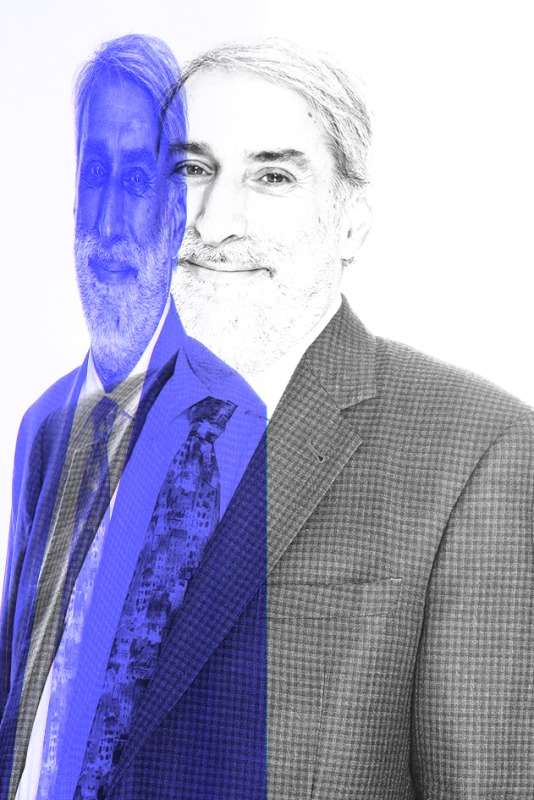
Editor-in-Chief Dan Nocera encourages the chemical science community to share the wealth of their most exceptional scientific discoveries, so that this is freely available to all.

The Giving Pledge is a moral commitment by some of the world's wealthiest individuals and families to contribute a majority of their wealth to philanthropic causes. It is a noble cause directed toward addressing society's most pressing problems. The pledge, and the people who have committed to the pledge, are to be admired for their generosity and vision. As scientists we typically do not accrue vast wealth in our lifetimes…but if we did, it is safe to say that the majority of scientists that I know would make The Giving Pledge. Notwithstanding, in the absence of financial fortune, scientists still hold a special type of wealth, their discovery. We often are quick to realize the impact of our discovery on a specific subject area of our scientific devotion and passion. Yet our discovery reaches beyond that immediacy. We are delighted to learn that our discovery “here” helped and contributed in a scientific discipline “over there”. Still deeper, have you ever thought about how your discovery may affect the young student that you never met? With the hesitation of dating myself, my enthusiasm for science was spurred by reading Encyclopedia Britannica. One could peruse, usually a page and never more than two, the “Wikipedia” of the day and learn about an obscure creature of organismic biology and then turn the page and be introduced to the cosmology of black holes. As a child, the wonders of science fell off those pages and set my course to pursue a career in science. As Editor-in-Chief of *Chemical Science*, I have had *déjà vu* moments when students and scientists from the smallest universities in India, Africa and Indonesia have thanked me for the availability of articles in *Chemical Science*, owing to the privileges offered by the Royal Society of Chemistry to maintain *Chemical Science* as a Gold Open Access journal, with all publication charges waived until at least the end of 2018. Immediately upon publication, the science of authors in *Chemical Science* is available to all on the web, free of charge. I often wonder what young mind is finding their calling to science through the discovery described on the pages of *Chemical Science.* More directly, I feel satisfied in the immediate engagement of the scientist, from a university with little resources available for subscriptions, to have access to forefront discovery. In this vein, the choice of an author to publish in *Chemical Science* makes a Giving Pledge by sharing their discovery by virtue of Gold Open Access. They are able to share their most precious wealth with the global community immediately, as their articles can be downloaded free from the web with no barriers to access. And they can do so with the freedom of an Edge article, which may be a short communication or a long full paper; Edge articles have no minimum or maximum length restrictions. With Gold Open Access and a publication waiver, also comes the responsibility to ensure we are selecting only research of the most exceptional significance from across the chemical sciences. I am thusly proud of the Associate Editors of *Chemical Science*, who take this charge seriously and strive to maintain the highest standard for the journal with a rigorous peer review process. So, as a scientist, take a Giving Pledge in 2017, and consider publishing your most extraordinary science for all to access in *Chemical Science*.
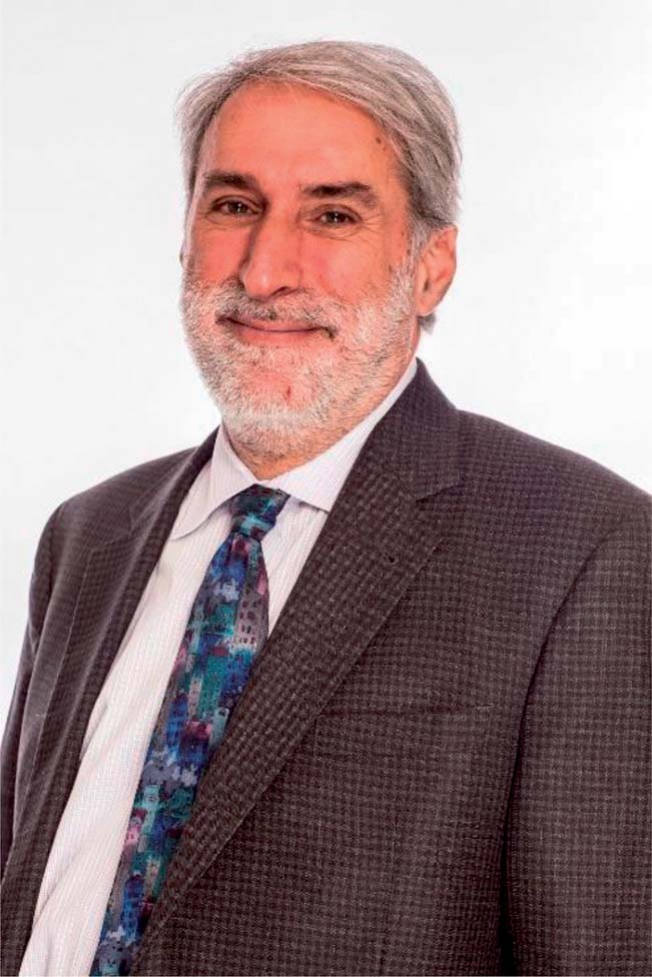


Professor Dan Nocera

Chemical Science, Editor-in-Chief

